# African walnut (*Plukenetia conophora*) oil promotes glucose uptake while improving energy metabolism and steroidogenesis and maintaining surface architecture in rat testes

**DOI:** 10.3389/fnut.2024.1505453

**Published:** 2024-11-19

**Authors:** Ochuko L. Erukainure, Chika I. Chukwuma

**Affiliations:** ^1^Laser Research Centre, Faculty of Health Sciences, University of Johannesburg, Doornfontein, South Africa; ^2^Centre for Quality of Health and Living, Faculty of Health and Environmental Sciences, Central University of Technology, Bloemfontein, South Africa

**Keywords:** African walnut, essential fatty acids, glucose metabolism, male fertility, steroidogenesis

## Abstract

**Background:**

African walnut (*Plukenetia conophora*) oil (AWO) has been reported for its nutritional and medicinal properties and has been employed for the management of metabolic diseases including hyperglycemia-mediated ailments.

**Objective:**

In the present study, AWO was investigated for its ability to stimulate glucose uptake and its effect on energy metabolism, steroidogenesis, and tissue morphology in isolated testes of Wistar rats.

**Methods:**

Isolated testes were incubated with AWO (30–240 μg/mL) in the presence of 11.1 mMol glucose at 37°C for 2 h. Control consisted of testes incubated with glucose only, while normal control consisted of testes not incubated with AWO and/or glucose. The standard antidiabetic drug was metformin.

**Results and conclusion:**

Incubation with AWO led to significant increase in glucose uptake, hexokinase, glyoxalase 1, glutathione reductase, glutathione peroxidase, 3β-hydroxysteroid dehydrogenase, 17β-hydroxysteroid dehydrogenase activities, GLUT4, glutathione, and ATP levels while concomitantly suppressing glucose-6-phosphatase, fructose-1,6-biphosphatase, glycogen phosphorylase, aldose reductase, polyol dehydrogenase, E-NTPDase, and ATPase activities. Furthermore, incubation with AWO led to improved testicular morphology while elevating testicular levels of magnesium, sulfur, potassium, calcium, and iron. Fatty acid profiling with GC-MS revealed linoleic acid and linolenic acid as the predominant essential fatty acids in AWO. Molecular docking analysis revealed potent molecular interactions of linoleic acid and linolenic acid with GLUT4. These results suggest the ability of AWO to improve testicular glucose metabolism and steroidogenesis and can be explored in the management of male infertility.

## 1 Introduction

The role of glucose metabolism in male fertility has been linked to its effect on the regulation and maintenance of spermatogenesis (1). This includes ATP production by anaerobic glycolysis and oxidative phosphorylation, essential for sperm motility, capacitation, and fertilization capability (2, 3). The testicular machinery system consists of glucose transporters which regulate glucose uptake and are sensitive to insulin, glucose-metabolizing enzymes, glycogen, and the blood–testis barrier (BTB) (1, 4, 5). This machinery tightly regulates testicular glucose metabolism, and its alteration has been linked to impaired glucose uptake and dysmetabolism, and reduced ATP level and oxidative stress, leading to impaired spermatogenesis and infertility (6). Thus, improving testicular glucose uptake and metabolism maybe a therapeutic strategy in the treatment of male infertility.

African walnuts are among the common underutilized nuts with reported nutritional and health benefits. Scientifically known as *Plukenetia conophora*, African walnuts belong to the Euphorbiaceae family and are widely distributed across sub-Saharan Africa (7). They are indigenous to tropical western and central Africa from Nigeria to Congo (8). They are grown mainly for their nuts which are utilized for food snacks. The nuts have been reported for their high oil content. The fatty acids and chemical constituents of the oil consist of 5-octadecenoic acid, methyl ester; tridecanoic acid, methyl ester; palmitic acid; 9-octadecenoic acid, methyl ester; oleic acid; and oleic anhydride (9). The oil has been shown to improve lipid metabolism by decreasing serum triglyceride and cholesterol, and LDL cholesterol levels while concomitantly decreasing the activities of alkaline phosphatase (ALP), aspartate aminotransferase (AST), and alanine aminotransferase (ALT) in liver and cardiac tissues of normal male albino rats (10). This effect has also been demonstrated in diabetes (11) and sodium arsenate induced toxicity, where it also attenuates oxidative stress by elevating antioxidant enzyme activities and quenching lipid peroxidation (12). Uhunmwangho and Omoregie (12) further demonstrated the ability of the oil to maintain hepatic morphology by preserving hepatic parenchyma, central vein, and hepatocytes. The antidiabetic properties of the oil have been demonstrated by its ability to reduce blood glucose level in people with type 2 diabetes (11).

Although the safety and health benefits of African walnut oil have been demonstrated, there is still a dearth on its effect on male fertility. Thus, the present study was carried out to investigate the ability of the oil to stimulate testicular glucose uptake and its effect on glucose metabolism and steroidogenesis.

## 2 Materials and methods

### 2.1 Plant material

African walnut fruits were purchased from a local fruit vendor at Ore, Ondo State, Nigeria. The fruits were washed with tap water and dehulled to obtain the seeds. The seeds were pulverized after air-drying, and 50 g of it was subjected to hexane (800 mL) extraction by immersing in hexane in a glass beaker at room temperature (25°C). After 3 days, the hexane extract was concentrated by exposing to air flow in a fume cupboard to obtain oil. The oil was stored in glass vials at room temperature (25°C) for further analyses.

### 2.2 Animals for *ex vivo* studies

Five male Wistar albino rats, weighing 180–250 g, were obtained and housed at the animal house facility of the Department of Biochemistry, College of Medicine, University of Lagos, Nigeria. The animals were euthanized with halothane after overnight fasting. Their testes were harvested and used immediately for *ex vivo* studies. The study was carried out under the approved protocol, CMUL/REC/00314.

### 2.3 Glucose uptake in isolated testicular tissues

Glucose uptake was determined using a previously described method (13). The choices of buffer pH and temperatures are based on the published method. In brief, 0.5 g of the freshly harvested testes was incubated with 8 mL of Krebs buffer, 11.1 mM glucose, and different concentrations of African walnut oil (30–240 μg/mL) for 2 h under a 5% CO_2_, 95% oxygen, and 37°C conditions. A reaction mixture incubated without the oil served as a normal control, while metformin served as the standard drug. Glucose concentrations of aliquots collected from the reaction mixtures before and after the incubation were measured with a Glucose (GO) Assay Kit (Merck, Johannesburg, South Africa) according to the manufacturer's manual. Glucose uptake was calculated using the formula:


$$\begin{array}{l} Glucose\;uptake\;per\;g\;of\;testis=\frac{GC1-GC2\;}{Weight\;of\;testicular\;tissue\;\left(g\right)} \end{array}$$

where GC1 and GC2 are glucose concentrations (mg/dL) before and after incubation, respectively.

After glucose uptake determination, ~1 mm was excised from the testicular tissues and fixed in 2.5% glutaraldehyde for electron microscopy analysis (14). The remaining tissues were homogenized in a cold 50 mM phosphate buffer solution (pH 7.5) containing 1% triton X-100. The homogenized tissues were centrifuged for 10 min at 15,000 rpm, 4°C. The supernatants were collected into 2 mL Eppendorf tubes and stored at −20°C until further biochemical analyses.

### 2.4 Hexokinase activity

Hexokinase activity in the tissues was determined using a hexokinase (HK) activity assay kit (Elabscience, Houston TX, USA) according to the manufacturer's instruction.

### 2.5 Glucogenic enzyme activities

The tissues were assayed for glucogenic enzyme activities which cover fructose-1,6-biphosphatase, glucose-6-phosphatase, and glycogen phosphorylase activities using previously published methods (15–19). The choices of buffer pH and temperatures are based on the published methods.

#### 2.5.1 Fructose-1,6-bisphosphatase activity

In brief, 100 μL of the tissue supernatant was mixed with 100 μL of 0.05 M fructose, 1,200 μL of 0.1 M Tris–HCl buffer (pH 7.0), 250 μL 0.1 M MgCl_2_, 100 μL 0.1 M KCl, and 250 μL 1 mM EDTA and incubated for 15 min at 37°C. Then, 10% TCA was added to stop the reaction mixture and centrifuged for 10 min at 3,000 rpm (4°C). Next, 50 μL of 1.25% ammonium molybdate and a freshly prepared 9% ascorbic acid were added to 100 μL of the resulting supernatant in a 96-well plate. The reaction mixture was allowed to stand for 20 min at room temperature (25°C). Absorbance was measured at 680 nm using a microplate reader (SpectraMax M2 microplate reader, Molecular Devices, San Jose, CA, USA).

#### 2.5.2 Glucose-6-phosphatase activity

In brief, 200 μL of the tissue supernatant was mixed with 100 μL of 0.25 M glucose-6-phosphatase, 200 μL of 5 mM KCl, and 1,300 μL of 0.1 M Tris-HCl buffer and incubated at 37°C in a shaker for 30 min. Next, 1 mL of distilled water and 1.25% ammonium molybdate were added to the reaction mixture to stop the reaction. This was followed by the addition of 1 mL of freshly prepared 9% ascorbate to the reaction mixture. The reaction mixture was allowed to stand for 30 min. Absorbance was then measured at 660 nm using a microplate reader (SpectraMax M2 microplate reader, Molecular Devices, San Jose, CA, USA).

#### 2.5.3 Glycogen phosphorylase activity

In brief, 100 μL of the tissue supernatant was mixed with 64 mM glucose-1-phosphate and 4% glycogen and incubated at 37°C for 10 min. Then, 20% ammonium molybdate in concentrated H_2_SO_4_ was added to reaction mixture to stop the reaction. Elon reducer and distilled water were then added to the reaction mixture and incubated for 45 min at 30°C. Absorbance was read at 340 nm using a microplate reader (SpectraMax M2 microplate reader, Molecular Devices, San Jose, CA, USA).

### 2.6 Polyol pathway

The polyol pathway was investigated in the testes by determining the aldose reductase and polyol dehydrogenase activities using previously published protocols (20, 21). The choices of buffer pH and temperatures are based on the published methods.

#### 2.6.1 Aldose reductase activity

In brief, 100 μL of the tissue supernatant was added to a reaction mixture of 700 μL of phosphate buffer (pH 6.7), 100 μL of 0.25 mM NADPH, and 100 μL of DL-glyceraldehyde at room temperature (25°C). Absorbance (OD) was read at 340 nm for 3 min at 30-s intervals using a microplate reader (SpectraMax M2 microplate reader, Molecular Devices, San Jose, CA, USA).

#### 2.6.2 Polyol dehydrogenase activity

In brief, 10 μL of the tissue supernatant was mixed to 240 μL of 50 mM glycine/NaOH buffer (pH 10.0) containing 57 mM sorbitol in a 96-well plate. Then, 50 μL of 50 mM NAD^+^ was added to the reaction mixture at room temperature (25°C). Absorbance was read at 340 nm for 3–5 min at 30-s intervals using a microplate reader (SpectraMax M2 microplate reader, Molecular Devices, San Jose, CA, USA).

### 2.7 Glyoxalase 1 activity

The glyoxalase 1 (GLO1) activity of the tissues was determined using a previously published protocol (22). In brief, 50 μL of 50 mM phosphate buffer (pH 6.6) was mixed with equal volumes of 2 mM methylglyoxal solution and 2 mM reduced glutathione (GSH). The reaction mixture was incubated for 30 min at 37°C. Thereafter, 10 μL of the tissue supernatant was added to the reaction mixture and further incubated for 10 min at 37°C. Absorbance was read 4 times at 240 nm at 2-min interval using a microplate reader (SpectraMax M2 microplate reader, Molecular Devices, San Jose, CA, USA).

### 2.8 Glutathione metabolism

Glutathione metabolism was determined in the tissues by assaying for reduced glutathione (GSH) level, glutathione reductase, and glutathione peroxidase activities according to previously published protocols (23–25). The choices of buffer pH and temperatures are based on the published methods.

#### 2.8.1 GSH level

In brief, 150 μL of the tissue supernatant was precipitated with equal volume of 10% trichloroacetic acid (TCA) and centrifuged at 2,000 rpm for 10 min at 25°C. Next, 80 μL of the supernatant was mixed with 40 μL of 0.5 mM DTNB in a 96-well plate. Then, 200 μL of 0.2M phosphate buffer (pH 7.8) was added to the reaction mixture and incubated at room temperature (25°C) for 15 min. Absorbance was measured at 415 nm using a microplate reader (SpectraMax M2 microplate reader, Molecular Devices, San Jose, CA, USA). The GSH level was extrapolated from a GSH standard graph.

#### 2.8.2 Glutathione reductase activity

In brief, 10 μL of the tissue supernatant was mixed with 221 μL of 50 mM Tris-HCl buffer (containing 1 mM EDTA, pH 8.0) and 38 μL of 8 mM oxidized glutathione (GSSG) in a 96-well plate at room temperature (25°C). Thereafter, 10 μL of NADPH was added to the reaction mixture. Absorbance was read at 340 nm for 8 min at a 2-min interval using a microplate reader (SpectraMax M2 microplate reader, Molecular Devices, San Jose, CA, USA).

#### 2.8.3 Glutathione peroxidase activity

In brief, 5 μL of the tissue supernatant was mixed with 210 μL of phosphate buffer (pH 6.9), 2.5 μL of 100 mM GSH, and 5 μL of distilled H_2_O in a 96-well plate and incubated for 10 min at 37°C. Thereafter, 2.5 μL of 15 mM NADPH was added to the reaction mixture, followed by 10 μL of 1 mM Ellman's reagent (Sigma, Johannesburg, South Africa). Absorbance was read at 412 nm using a microplate reader (SpectraMax M2 microplate reader, Molecular Devices, San Jose, CA, USA). The activity was extrapolated from a GSH standard graph.

### 2.9 Lipid peroxidation

Lipid peroxidation was determined in the tissues by assaying for malondialdehyde (MDA) level using previously published protocol (26). In brief, 200 μL of the tissue supernatant was mixed with 200 μL of 8.1% SDS solution, 750 μL of 20% acetic acid, 2 mL of 0.25% thiobarbituric acid (TBA), and 850 miliQ water. The reaction mixture was boiled for 1 h. After cooling to room temperature (25°C), a 200 μL aliquot of the reaction mixture was pipetted into a 96-well plate, and the absorbance was measured at 532 nm using a microplate (SpectraMax M2 microplate reader, Molecular Devices, San Jose, CA, USA). Lipid peroxidation was estimated from the concentrations of thiobarbituric acid reactive substances (TBARS) extrapolated from the MDA standard curve.

### 2.10 Nucleotide metabolism

Nucleotide metabolism was determined in the tissues by assaying for ATP level, E-NTPDase, and ATPase activities in previously published methods (27, 28).

#### 2.10.1 ATP level

In brief, 50 μL of the tissue supernatant was mixed with equal volume of CellTiter-Glo^®^ reagent (Madison, WI, USA) in an opaque 96-well plate. The reaction mixture was incubated in the dark for 30 min on a shaker at 25°C. Luminescence was measured at room temperature (25°C) using a microplate reader (SpectraMax M2 microplate reader, Molecular Devices, San Jose, CA, USA) according to the reagent manufacturer's protocol.

#### 2.10.2 E-NTPDase activity

In brief, 20 μL of the tissue supernatant was mixed with 200 μL of the reaction buffer (1.5 mM CaCl_2_, 5 mM KCl, 0.1 mM EDTA, 10 mM glucose, 225 mM sucrose, and 45 mM Tris-HCl) and incubated at 37°C for 10 min. Next, 20 μL of 50 mM ATP was added to the reaction mixture and further incubated in a shaker for 20 min at 37°C. Then, 200 μL of 10% TCA was used in terminating the reaction. Thereafter, 200 μL of 1.25% ammonium molybdate and a freshly prepared 9% ascorbic acid was added to the reaction mixture. The reaction mixture was allowed to stand on ice for 10 min, and absorbance was read at 600 nm using a microplate reader (SpectraMax M2 microplate reader, Molecular Devices, San Jose, CA, USA).

#### 2.10.3 ATPase activity

In brief 200 μL of the tissue supernatant was mixed with 200 μL of 5 mM KCl, 1,300 μL of 0.1 M Tris-HCl buffer, and 40 μL of 50 mM ATP and incubated in a shaker for 30 min at 37°C. The reaction was stopped by the addition of 1 mL of a distilled water and ammonium molybdate to the reaction mixture. A freshly prepared 9% ascorbic acid was added to the mixture and allowed to stand on ice for 10 min. Absorbance was measured at 660 nm using a microplate reader (SpectraMax M2 microplate reader, Molecular Devices, San Jose, CA, USA).

### 2.11 Glucose transporter 4 level

GLUT4 level in the tissues was determined using a GLUT4 ELISA Assay Kit (Merck, Johannesburg, South Africa) according to the manufacturer's instruction.

### 2.12 Steroidogenic enzyme activities

Steroidogenic enzyme activities were determined in the tissues by assaying for the activities of 3-beta-hydroxysteroid dehydrogenase (3β-HSD) and 17-beta-hydroxysteroid dehydrogenase (17-β-HSD) according to previously published methods (29, 30). The choices of buffer pH and temperatures are based on the published methods.

#### 2.12.1 3β-HSD activity

In brief, 50 μL of the tissue supernatant was mixed with 25 μL of 100 mM sodium pyrophosphate buffer (pH 8.9), 10 μL ethanol containing 0.3 mM dihydroxyl epiandrosterone (DHEA), and 40 μL of 25% bovine serum albumin (BSA). Then, 50 μL of 0.5 mM NAD^+^ was added to the reaction mixture at room temperature (25°C). Absorbance was read at 340 for 8 min at 2-min interval using a microplate reader (SpectraMax M2 microplate reader, Molecular Devices, San Jose, CA, USA).

#### 2.12.2 17-β-HSD activity

In brief, 50 μL of the tissue supernatant was mixed with 25 μL of 100 mM sodium pyrophosphate buffer (pH 10.2), 10 μL ethanol containing 0.3 mM testosterone (DHEA), and 40 μL of 25% bovine serum albumin (BSA). Then, 50 μL of 0.5 mM NAD^+^ was added to the reaction mixture. Absorbance was read at 340 for 8 min at 2-min interval using a microplate reader (SpectraMax M2 microplate reader, Molecular Devices, San Jose, CA, USA).

### 2.13 Microscopic analysis

The excised tissues were subjected to scanning electron microscopy (SEM) and EDX-SEM analyses to determine their morphology and elemental composition using previous published protocols (14). The choices of buffer pH and temperatures are based on the published method. In brief, the fixed tissues were subjected to buffer (50 mM phosphate buffer pH 7.0) wash to remove glutaraldehyde before post-fixed with 0.5% osmium tetroxide for 2 h. After washing with distilled water, the tissues were dehydrated with ethanol of increasing concentrations: 25% (twice at 5-min interval), 50% (twice at 5-min interval), 75% (twice at 5-min interval), and 100% (twice at 10-min interval). A critical-point-dryer apparatus was utilized in drying the tissue. They were gold-coated and observed with a SEM (Zeiss Ultra Plus) at an accelerating voltage of 20–25 kV.

### 2.14 Gas chromatography–mass spectrometry analysis

To determine the fatty acid constituents of the extracted oil, the oil was subjected to GC-MS analysis using previously established protocol and operating conditions (31). The analysis was carried out with an Agilent technologies 6890 series GC coupled with (an Agilent) 5973 Mass Selective Detector and driven by Agilent ChemStation software. The operating parameters were as follows:

**Column**: HP-5MS capillary column (30 m × 0.25 mm ID, 0.25 μm film thickness, 5% phenylmethylsiloxane); **Carrier gas**: ultra-pure helium; **Flow rate**: 1.0 mL min^−1^ and a linear velocity of 37 cm s^−1^; **Injector temperature**: was set at 250°C. Initial oven temperature: 60°C, programmed to 280°C at the rate of 10°C min^−1^. **Injection**: 1 μL made in split mode at a split ratio of 20:1; **Electron ionization mode**: 70 eV; **Electron multiplier voltage**: at 1,859 V; **Ion source temperature**: 230°C; **Quadrupole temperature**: 150°C; **Solvent delay**: 4 min; **Scan range**: 50–70 amu. Fatty acids were identified by comparing the retention times and mass spectral data directly with those in an in-built National Institute of Standards and Technology (NIST) library database.

### 2.15 Molecular docking analysis

To understand the molecular interactions of the identified fatty acids with GLUT4, molecular docking analysis was carried out.

#### 2.15.1 Protein target selection and preparation

The active site amino acid sequence of GLUT4 protein (P19357) was retrieved from https://www.uniprot.org/, and the structure was modeled with https://swissmodel.expasy.org/. The protein was prepared and refined using Discovery Studio 2021 (https://discover.3ds.com/discovery-studio-visualizer-download) for docking. This was accomplished by removing the co-crystallized ligand and extra water molecules to transform the protein into a nascent receptor and then adding hydrogen and charges.

#### 2.15.2 Ligand selections and preparations

The structures of the most predominant compounds were modeled and their energy were optimized (MM44D) using Chem 3D 20.1 (https://perkinelmer-chemoffice-suite.software.informer.com/20.1/). These optimized structures were used in the molecular docking studies.

#### 2.15.3 Molecular docking

The molecular docking analysis was done on the compounds with the protein receptors using the Lamarckian Genetic Algorithm in AutoDock 4.2 (https://autodock.scripps.edu). The chosen ligand was docked with the active sites GLUT4 using Python Prescription 0.8 and a package of automated molecular docking tools called Auto Dock Vina 4.2. PDB files that had already been created as inputs were used to generate the protein's protein data bank, partial charge, and atom type a file (PDBQT). With the aid of a grid box, the enzyme's precise target location was established for the active site the receptor. The number of grid points X, Y, and Z dimensions were 34, 54, and 32, respectively, while the grid center's X, Y, and Z were 100.436, 105.26, and 107.614, respectively. To manual comparative analysis, 10 configurations for each protein–ligand complex were generated after the docking experiments for all the compounds. Text files with the dock scoring values were also prepared. The optimal docking pose was determined to be the conformation with the lowest binding energy (kcal/mol). Investigations on the interactions between ligands and proteins were also conducted, and the visual representation of the resulting conformations was created using Discovery Studio 2021.

### 2.16 Statistical analysis

All biological analyses were carried out in triplicates and subjected to statistical analysis using SPSS version 27. Data were subjected to one-way ANOVA and reported as mean ± SD. Data were considered significant at a *p*-value of < 0.05.

## 3 Results and discussion

Glucose plays important roles in testicular function and spermatogenic activities contributing to male fertility (32). The alterations of these functions and activities have been implicated in testicular dysfunction and, subsequently, infertility if not well-managed. African walnut and its oil have been employed in traditional medicine to treat various ailments including the management of glucose dysfunction-related diseases (11). In the present study, we investigated the effect of African walnut oil on glucose uptake and metabolism in rat testicular tissues. The oil yield was 45% which correlates with previous reports on the oil yield (33, 34).

### 3.1 Glucose uptake

**Figure 1:**
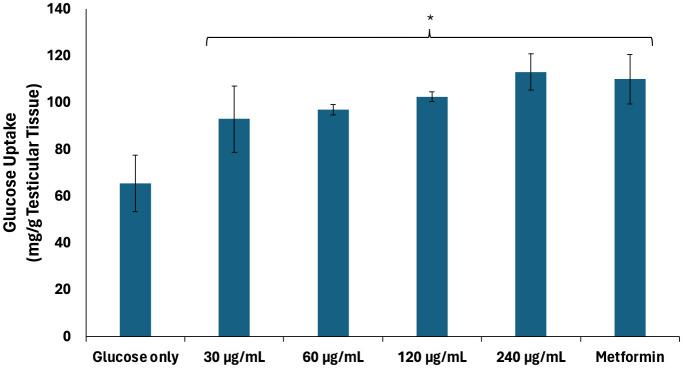
Effect of African walnut oil on testicular glucose uptake. Values = mean ± SD; *n* = 3. *Statistically significant (*p* < 0.05) to glucose only.

Testicular glucose uptake plays an important role on glucose bioavailability for energy metabolism required for gamete production, sperm nourishment and maturation, sperm capacitation, and spermatogenesis regulation (35, 36). As shown in Figure 1, African walnut oil significantly (*p* < 0.05) stimulated testicular glucose uptake dose-dependently, with the highest dose (240 μg/mL) comparing favorably with metformin. The glucose uptake activity of the oil indicates its potential in enhancing glucose bioavailability for testicular energy metabolism.

### 3.2 Glycolytic and glucogenic enzyme activities

**Figure 2:**
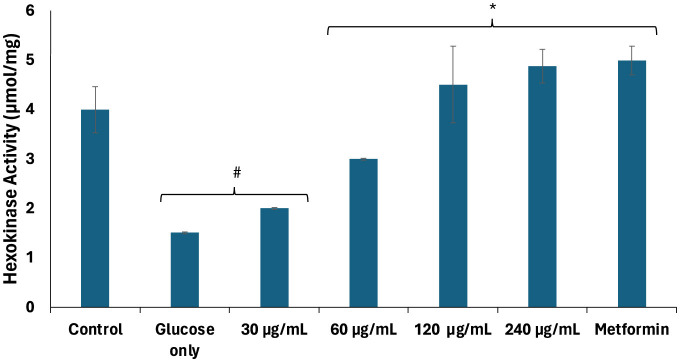
Effect of African walnut oil on hexokinase activity in testicular glucose uptake. Values = mean ± SD; *n* = 3. *Statistically significant (*p* < 0.05) to glucose only; ^#^statistically significant (*p* < 0.05) to control.

Impaired testicular glucose uptake has been linked to alterations in glycolytic enzyme activities in Sertoli cells, leading to suppressed lactate production and, subsequently, altered energy metabolism for spermatogenic activities (4, 36). As shown in Figure 2, there was a significant (*p* < 0.05) decrease in hexokinase activity of testicular tissues incubated with glucose only. Hexokinases regulate glucose metabolism by catalyzing the phosphorylation of glucose to glucose-6-phosphate, the rate-limiting first step of glycolysis (37). Thus, the decreased hexokinase activity indicates suppression of the glycolytic pathway. The activity was significantly (*p* < 0.05) elevated dose-dependently following incubation with African walnut oil which indicates activation of the glycolytic pathway. This also suggests increase in the production of lactate and improved energy metabolism for spermatogenic activities.

**Figure 3:**
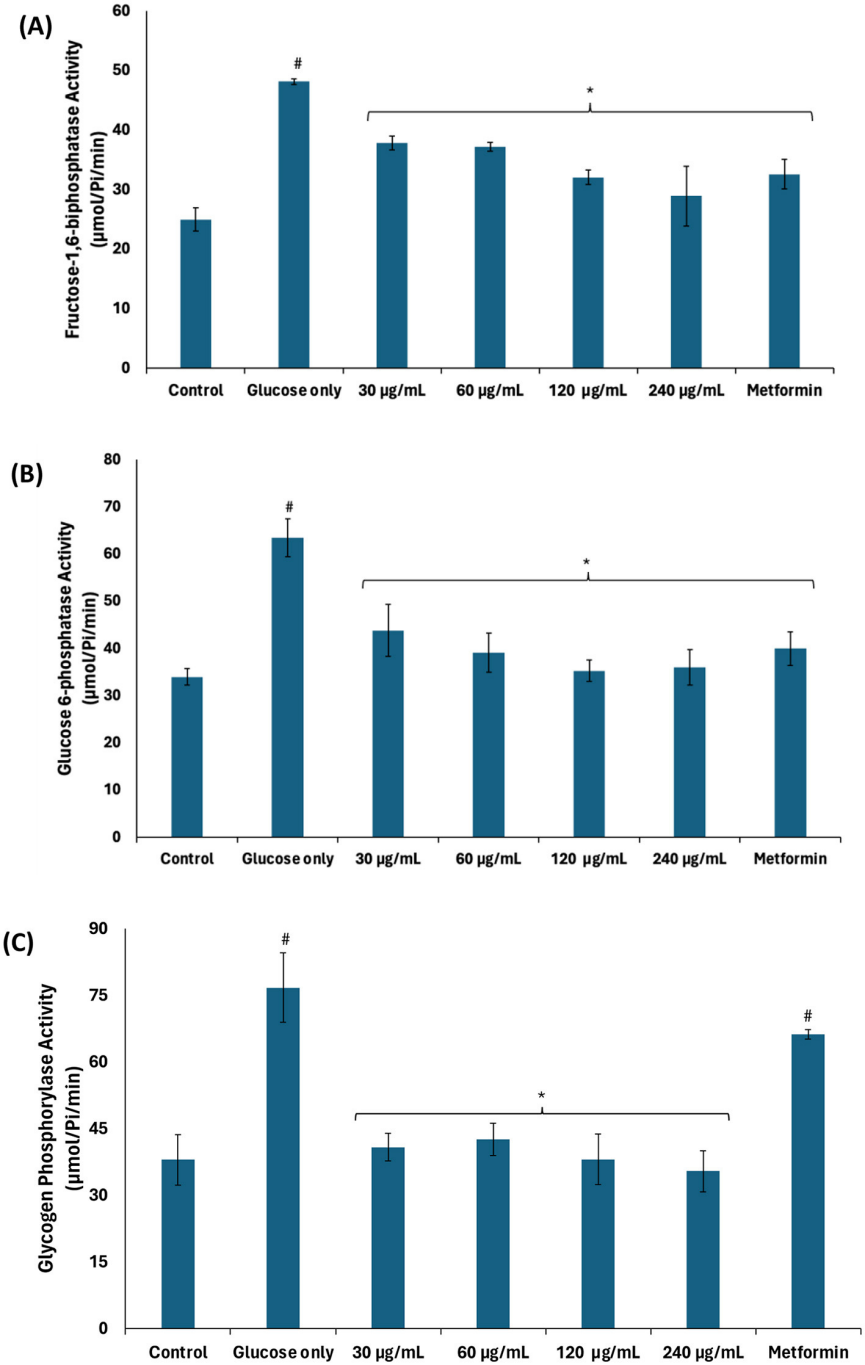
Effect of African walnut oil on glucogenic enzyme activities in testicular glucose uptake. **(A)** Fructose-1,6-biphosphatase; **(B)** glucose-6-phosphatase; and **(C)** glycogen phosphorylase. Values = mean ± SD; *n* = 3. *Statistically significant (*p* < 0.05) to glucose only; ^#^statistically significant (*p* < 0.05) to control.

The significant (*p* < 0.05) elevated activities of fructose-1,6-biphosphatase, glucose-6-phosphatase, and glycogen phosphorylase in testicular tissues incubated with glucose only (Figures 3A–C) suggest glucogenesis and glycogenolysis, thus further corroborating suppressed glycolysis. These activities were significantly (*p* < 0.05) suppressed dose-dependently following incubation with African walnut oil, thereby indicating suppressed glucogenesis and activation of the glycogenic and glycolytic pathways.

### 3.3 Polyol pathway

**Figure 4:**
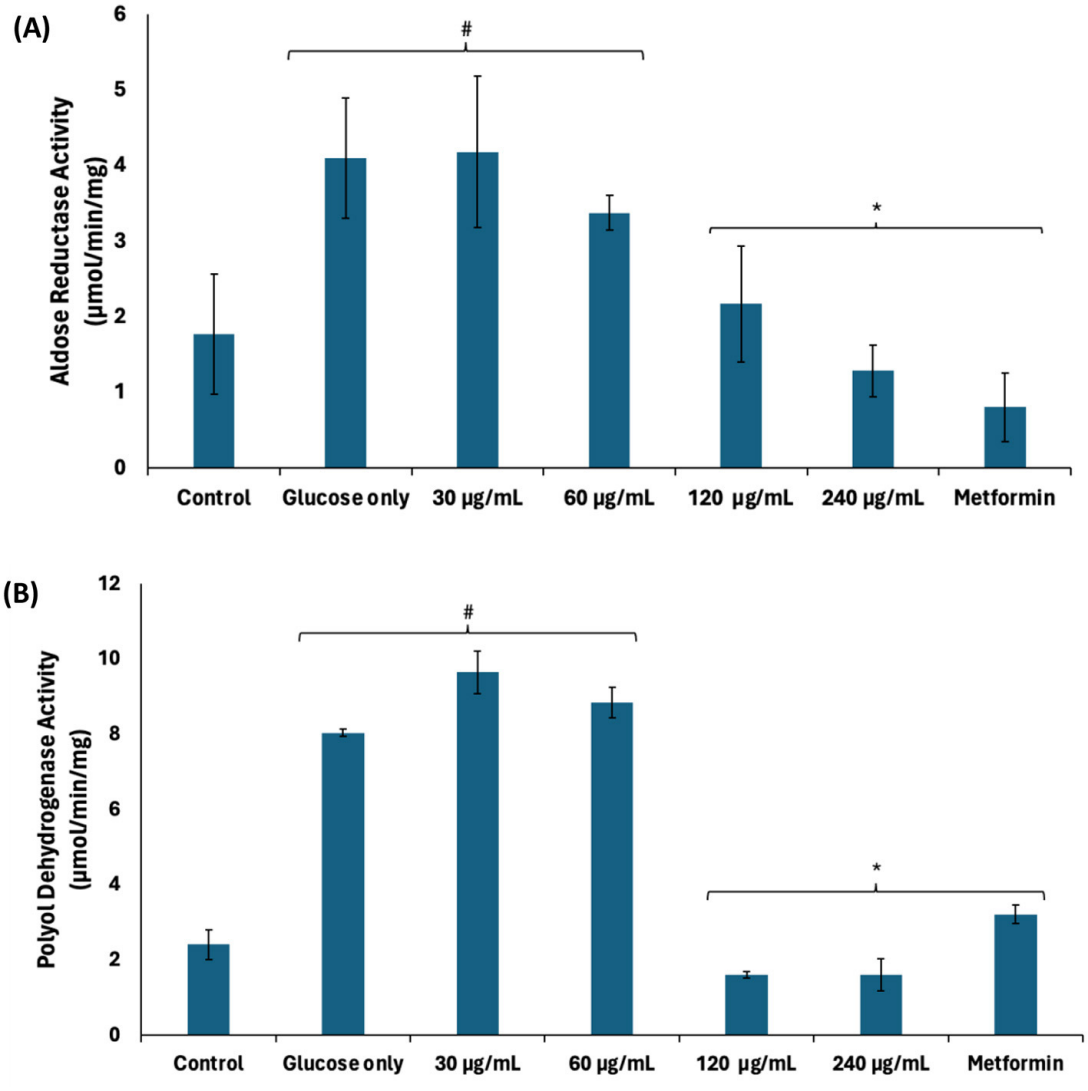
Effect of African walnut oil on polyol pathway in testicular glucose uptake. **(A)** Aldose reductase and **(B)** polyol dehydrogenase. Values = mean ± SD; *n* = 3. *Statistically significant (*p* < 0.05) to glucose only; ^#^statistically significant (*p* < 0.05) to control.

Continuous glucogenesis and glycogenolysis have been implicated in the continuous production of glucose leading to exacerbated cellular levels (38). Exacerbated cellular glucose levels may be channeled to other pathogenic pathways such as the polyol, hexosamine, AGE, and protein kinase C pathways leading to generation of toxic metabolites. These pathways have been reported as a major patho-mechanism of hyperglycemia-mediated spermatogenic disruption (39, 40). As shown in Figures 4A, B, there was a significant (*p* < 0.05) increase in the activities of aldose reductase and polyol dehydrogenase in testicular tissues incubated with glucose only. Aldose reductase catalyzes the conversion of glucose to sorbitol, the first step in the polyol pathway (41). The produced sorbitol is converted to fructose by polyol dehydrogenase. Thus, the elevated enzyme activities indicate channeling of excess of glucose arising from continuous glucogenesis and glycogenolysis into the polyol pathway. The enzyme activities were significantly (*p* < 0.05) suppressed dose-dependently following incubation with African walnut oil, thus suggesting suppressed production of fructose.

### 3.4 Glyoxalase system

**Figure 5:**
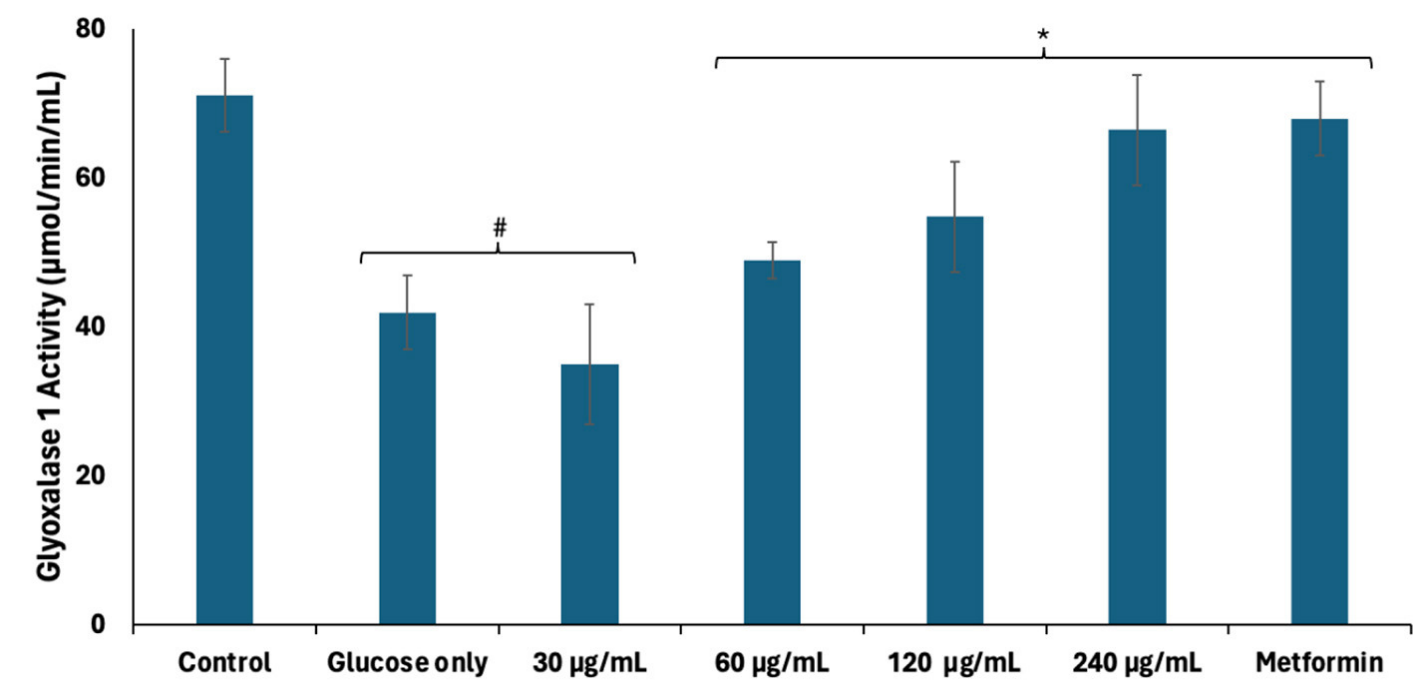
Effect of African walnut oil on glyoxalase 1 activity in testicular glucose uptake. Values = mean ± SD; *n* = 3. *Statistically significant (*p* < 0.05) to glucose only; ^#^statistically significant (*p* < 0.05) to control.

Accumulated fructose arising from continuous activation of the polyol pathway has been linked to the production of AGEs and has been implicated in hyperglycemia-mediated spermatogenic disruption (39). Excessive cellular fructose undergoes phosphorylation and hydrolysis to generate the triose, dihydroxyacetone phosphate (DHAP) which is further converted to the toxic metabolite, methylglyoxal (42). Methylglyoxal modifies and alters the functions of proteins, DNA, and lipids via glycation (42). Methylglyoxal is detoxified by converting to lactate via the glyoxalase system consisting of GLO1 which catalyzes the conversion of methylglyoxal to S-D-lactoylglutathione, and GLO2 which converts S-D-lactoylglutathione to lactate (43). As shown in Figure 5, GLO1 activity was significantly (*p* < 0.05) suppressed in testicular tissues incubated with glucose only, thus suggesting an increased accumulation of methylglyoxal and glycation, and correlates with the activation of the polyol pathway. Incubation with African walnut oil led to significant (*p* < 0.05) elevation in the activity of GLO1 dose-dependently, thereby suggesting reduced testicular level of methylglyoxal and, subsequently, an arrest of AGE production.

### 3.5 Glutathione metabolism and lipid peroxidation

**Figure 6:**
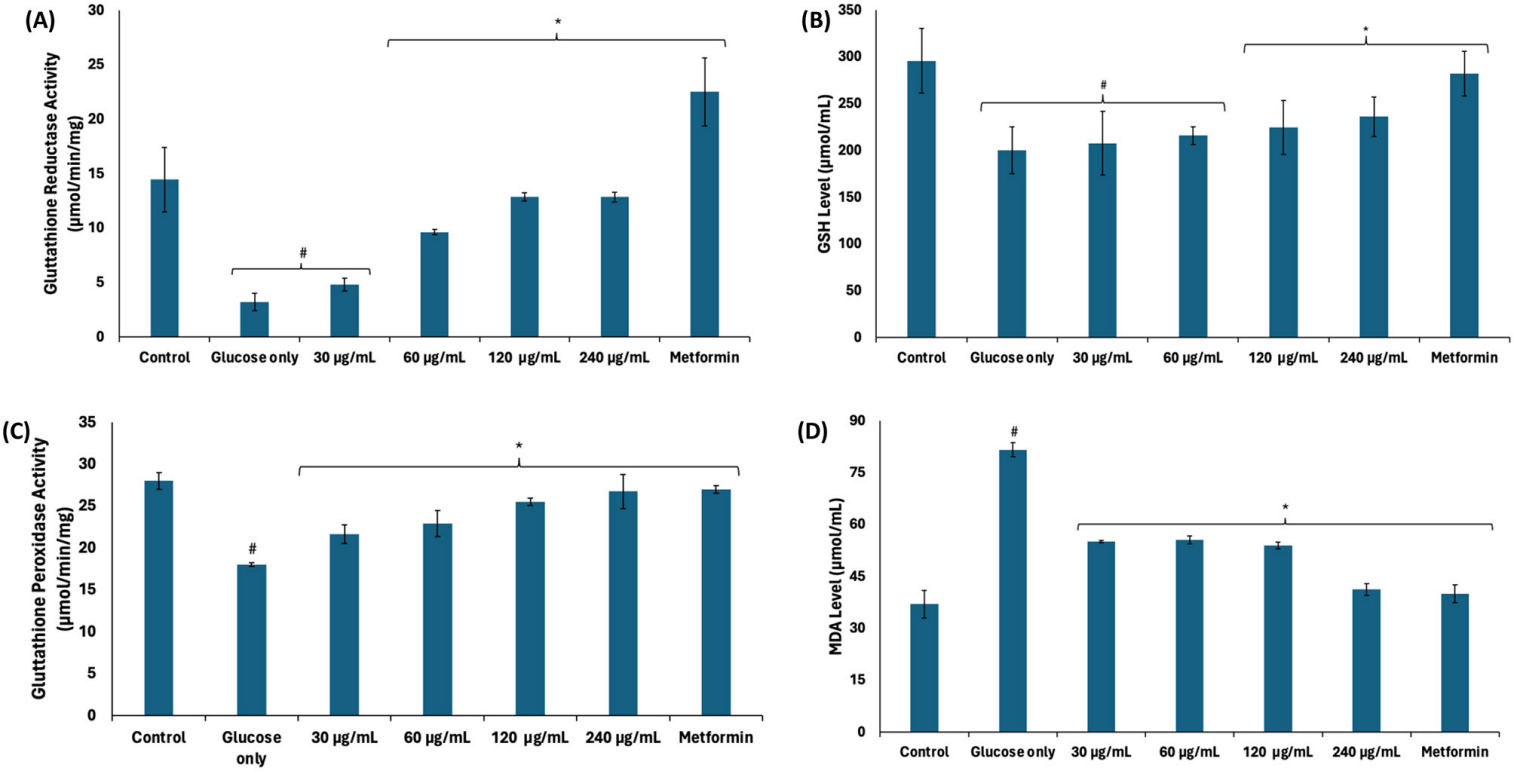
Effect of African walnut oil on **(A)** glutathione reductase; **(B)** GSH level; **(C)** glutathione peroxidase activities; and **(D)** MDA level in testicular glucose uptake. Values = mean ± SD; *n* = 3. *Statistically significant (*p* < 0.05) to glucose only; ^#^statistically significant (p < 0.05) to control.

Reduced glutathione (GSH) plays an important role in the glyoxalase system as it is a cofactor for GLO1 (44). As shown in Figures 6A–C, there was a significant (*p* < 0.05) decrease in glutathione reductase activity, GSH level, and glutathione peroxidase activity in testicular tissues incubated with glucose only. Glutathione reductase catalyzes the reduction of oxidized glutathione (GSSG) to GSH, while glutathione peroxidase catalyzes the oxidation of GSH to GSSG (45), while reducing hydrogen peroxide (H_2_O_2_) (46). A balance in the activities of both enzymes maintains constant cellular levels of GSH required for the glyoxalase system, antioxidant activities, and other biological activities. The reduced activities of these enzymes corroborate with the decreased GSH and further correlate with the decreased GLO1 activity. There was also an increase in testicular MDA level following incubation with glucose only (Figure 6D), indicating an occurrence of lipid peroxidation. This can be attributed to decreased glutathione peroxidase activity leading to increased cellular level of H_2_O_2_. Due to its instability, H_2_O_2_ decomposes to yield the hydroxyl radical, ·HO, which attacks lipid membranes to trigger peroxidative reactions (47). Incubation with African walnut oil significantly (*p* < 0.05) elevated the activities of glutathione reductase and glutathione peroxidase, and GSH level while suppressing MDA level, thus indicating the potential of the oil to maintain cellular GSH homeostasis for biological activities and arrest lipid peroxidation.

### 3.6 Nucleotide metabolism

**Figure 7:**
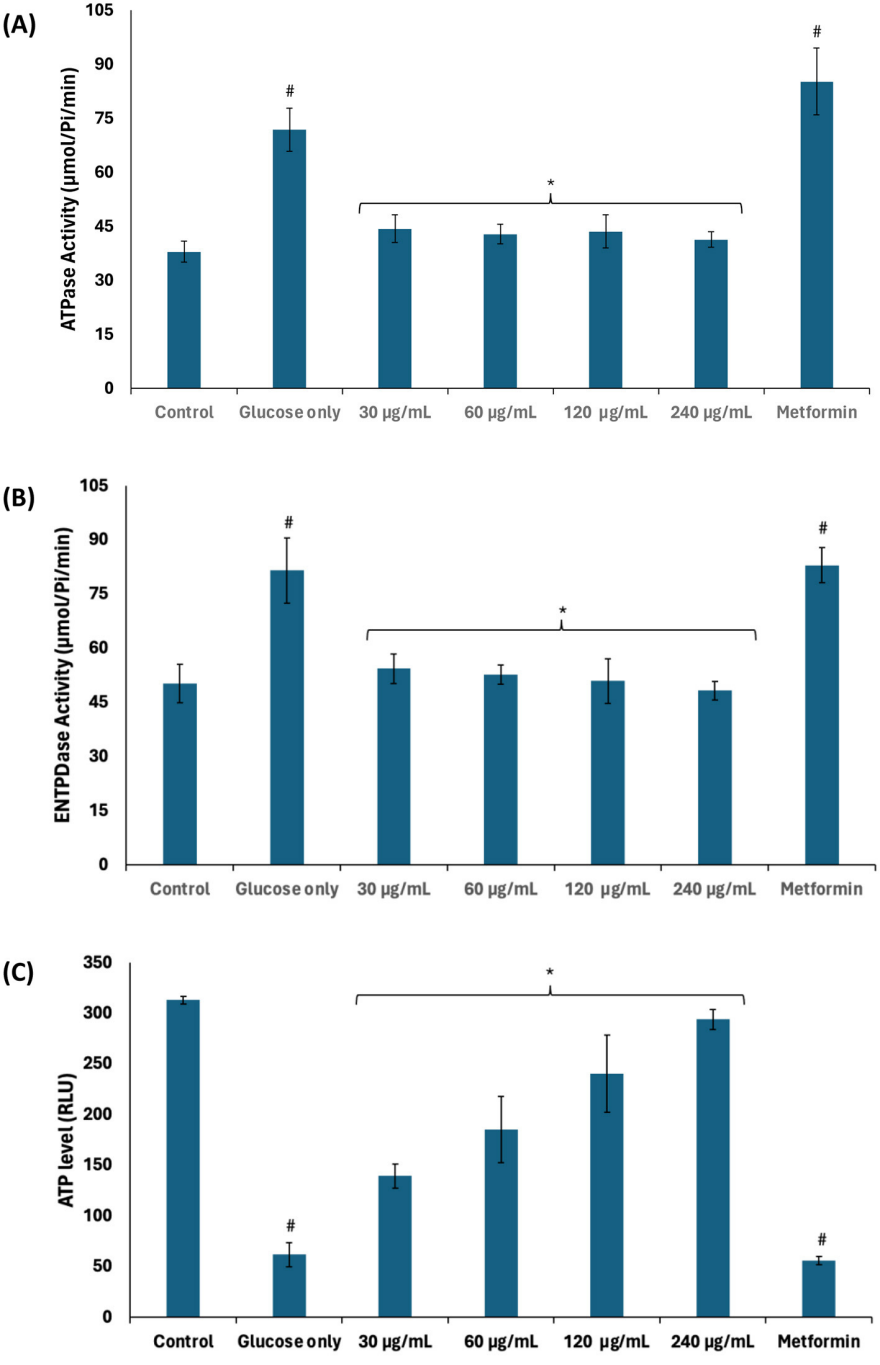
Effect of African walnut oil on nucleotide metabolism in testicular glucose uptake. **(A)** ATPase; **(B)** E-NTPDase activities; and **(C)** ATP level. Values = mean ± SD; *n* = 3. *Statistically significant (*p* < 0.05) to glucose only; ^#^statistically significant (*p* < 0.05) to control.

Nucleotide metabolism leading to the production and utilization of energy in the form of ATP is crucial to testicular functions such as germ cell development and spermatogenic activities (32, 36). Glycolytic phosphorylation leading to lactate production has been reported as the preferred source of ATP production for spermatogenic activity due to active glycolysis over the Krebs cycle (3, 36). As shown in Figures 7A–C, the activities of ATPase and E-NTPDase were significantly (*p* < 0.05) elevated in testicular tissues incubated with glucose only, with concomitant decreased ATP level. The decreased ATP level maybe attributed to the increased activities of ATPase and E-NTPDase. These enzymes catalyze the hydrolysis of ATP which reduces the availability of ATP for sperm capacitation, fertilization potential, and motility (2). The suppressed glycolysis and active glucogenesis may also contribute to the reduced ATP level. The enzyme activities and ATP level were significantly (*p* < 0.05) reversed following incubation with African walnut oil, thus suggesting the potential of the oil to improve testicular ATP levels. This is corroborated by the decreased ATPase and E-NTPDase activities as well as the active glycolytic and inactivated deactivated glucogenic activities. Glycolysis also produces two molecules of NADH which are channeled to the electron transport chain to further produce six molecules of ATP (48).

### 3.7 Glucose transporter

**Figure 8:**
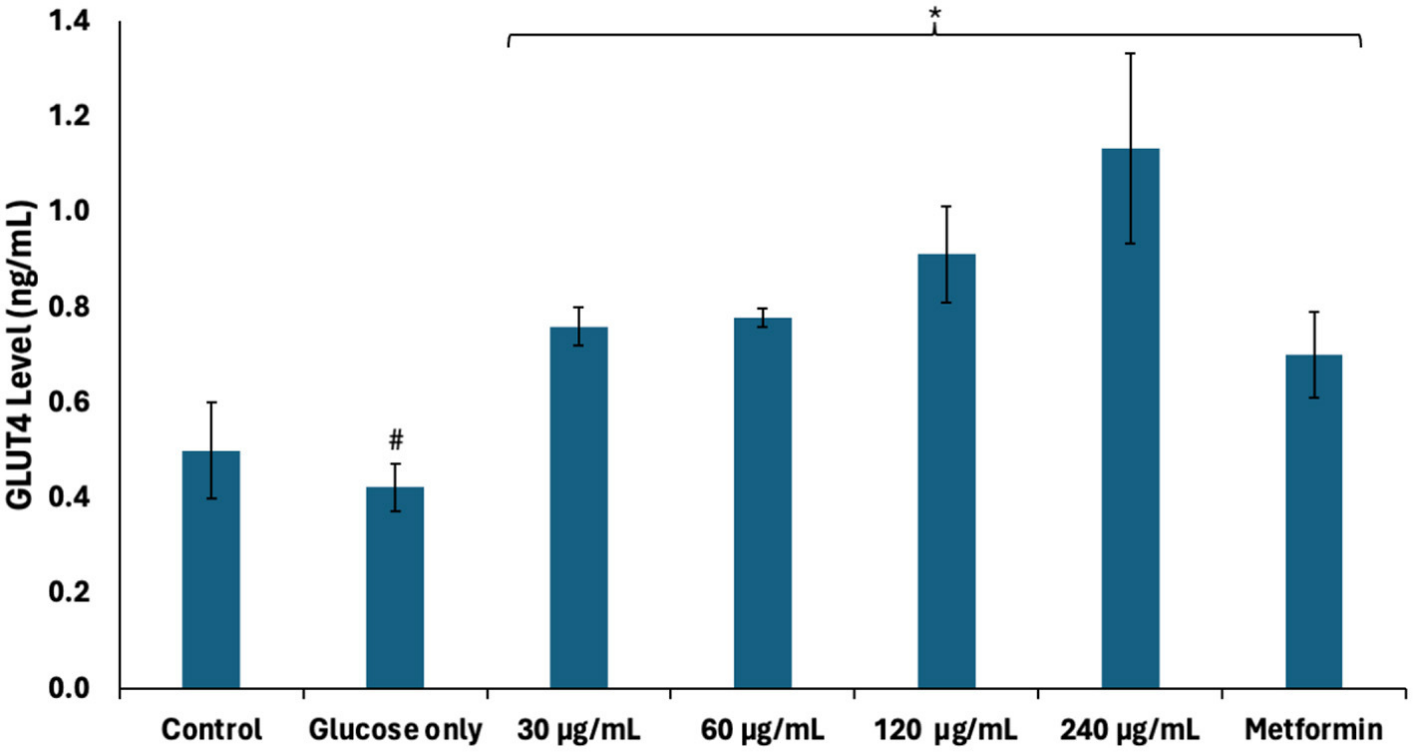
Effect of African walnut oil on GLUT4 level in testicular glucose uptake. Values = mean ± SD; *n* = 3. *Statistically significant (*p* < 0.05) to glucose only; ^#^statistically significant (*p* < 0.05) to control.

The role of glucose transporters (GLUTs) in regulating testicular glucose metabolism and energy production has been reported (36). They facilitate glucose uptake from the interstitial fluid into testicular tissues where they undergo glycolysis to yield ATP and NADH for energy metabolism required for spermatogenic activities (32, 36). As shown in Figure 8, GLUT4 level was significantly depleted (*p* < 0.05) in testicular tissues incubated with glucose only. This correlates with the suppressed glucose uptake and indicates decreased availability of glucose for glycolytic production of ATP and NADH. Incubation with African walnut oil significantly (*p* < 0.05) elevated testicular GLUT4 levels dose-dependently. This further indicates the potential of the oil to facilitate testicular glucose uptake and utilization for spermatogenic activities.

### 3.8 Steroidogenesis

**Figure 9:**
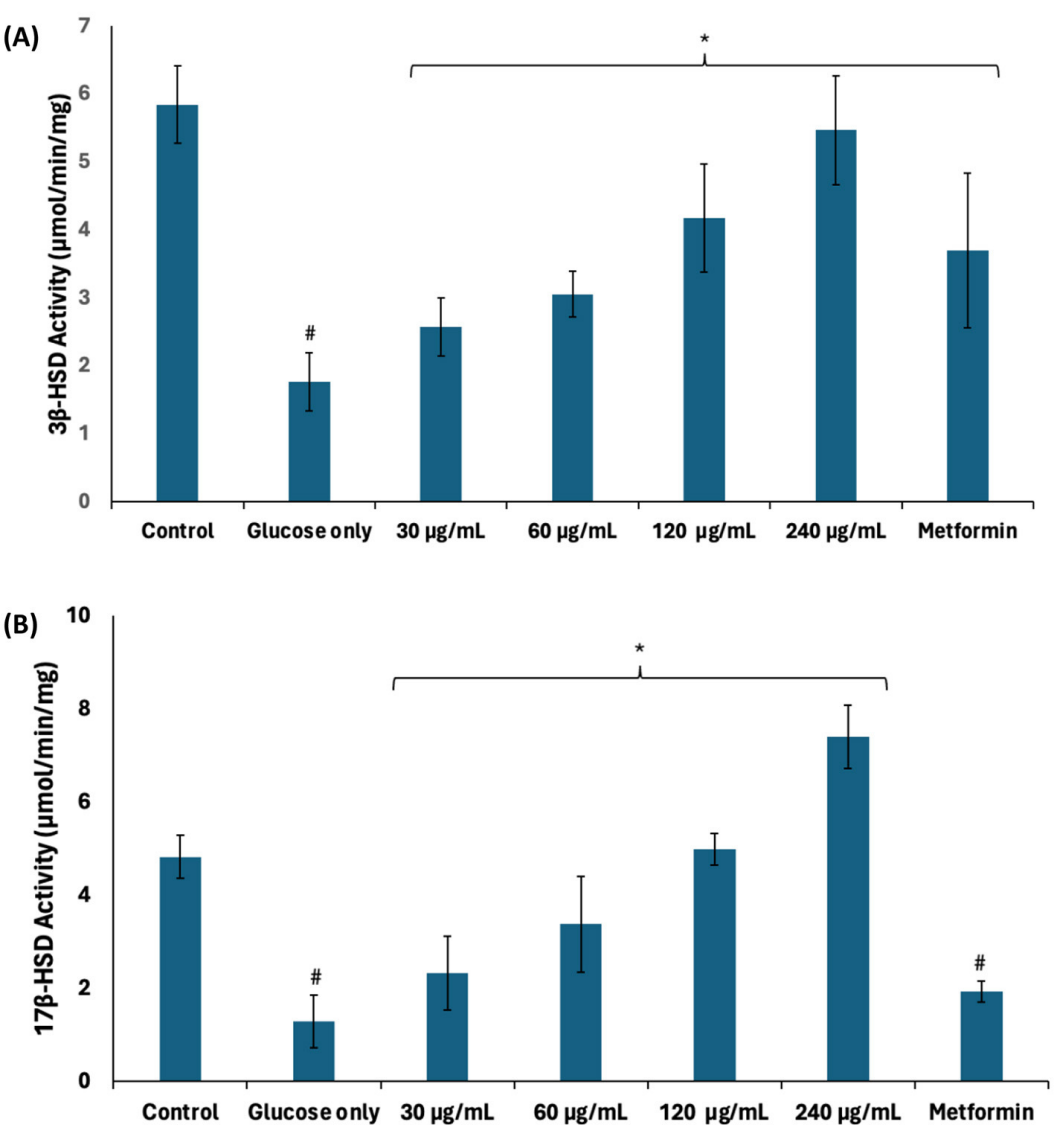
Effect of African walnut oil on steroidogenesis in testicular glucose uptake. **(A)** 3β-HSD and **(B)** 17β-HSD activities. Values = mean ± SD; *n* = 3. *Statistically significant (*p* < 0.05) to glucose only; ^#^statistically significant (*p* < 0.05) to control.

Steroidogenesis plays an important role in spermatogenesis as it produces testosterone which facilitates meiosis, differentiation, and maturation of germ cells (49). In steroidogenesis pathway, 3β-HSD catalyzes the conversion of dehydroepiandrosterone into the direct precursor of testosterone, androstenedione (4-dione), which is further converted into testosterone by 17β-HSD (50). There was a significant (*p* < 0.05) decrease in the activities of 3β-HSD and 17β-HSD in testicular tissues incubated with glucose only as shown in Figure 9. These decreases depict suppression of steroidogenesis and, subsequently, spermatogenesis. Steroidogenesis is energy-dependent, and it is altered in impaired testicular glucose homeostasis (32). 3β-HSD and 17β-HSD activities were significantly (*p* < 0.05) elevated following incubation with African walnut oil, thus indicating the potential of the oil to stimulate steroidogenesis and thus promote spermatogenesis.

### 3.9 Microscopic analysis

**Figure 10:**
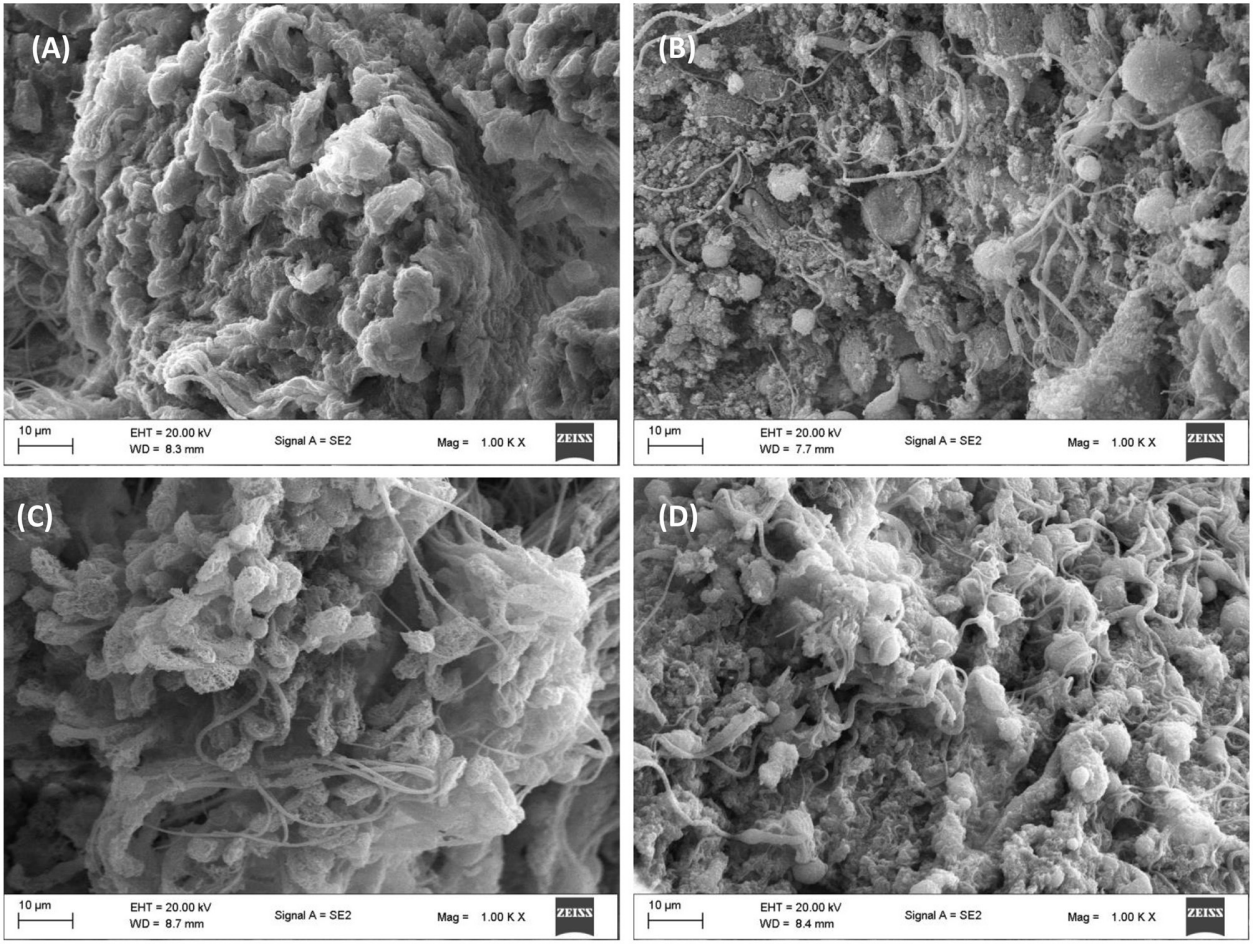
Effect of African walnut oil on tissue surface morphology in testicular glucose uptake. Magnification: 1000X. **(A)** control; **(B)** glucose only; **(C)** African walnut oil; and **(D)** metformin.

As shown in Figure 10A, surface view of testicular tissues revealed a stable tissue architecture depicted by well-preserved seminiferous tubules, with spermatids covering the surfaces. This architecture was distorted in testicular tissues incubated with glucose only (Figure 10B). Impaired glucose metabolism has been implicated in the distortion of testicular architecture and has been linked to perturbed spermatogenic activities (51). The tissue architecture was restored following incubation with African walnut oil (Figure 10C) and metformin (Figure 10D).

### 3.10 Elemental mapping

**Figure 11:**
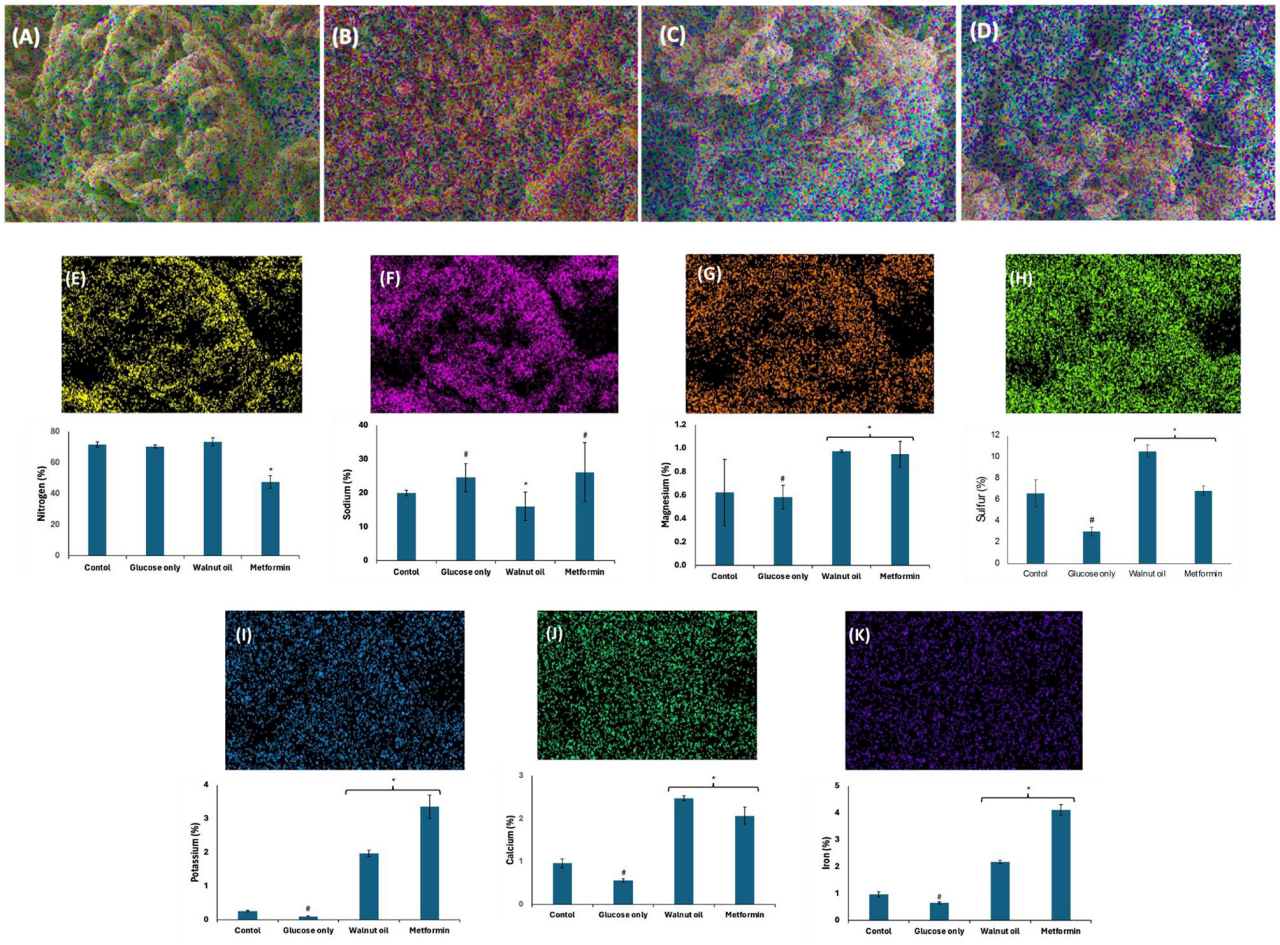
Effect of African walnut oil on tissue elemental constituents in testicular glucose uptake. Values = mean ± SD; *n* = 3. Magnification: 1000X. **(A)** control; **(B)** glucose only; **(C)** African walnut oil; **(D)** metformin; **(E)** nitrogen; **(F)** sodium; **(G)** magnesium; **(H)** sulfur; **(I)** potassium; **(J)** calcium; and **(K)** iron. *Statistically significant (*p* < 0.05) to glucose only; # statistically significant (*p* < 0.05) to control.

The role of elements in testicular glucose metabolism and spermatogenic activities including spermatogenesis, sperm maturation, and motility has been reported (52, 53). These elements are often co-factors for most enzymes involved in glucose metabolism and spermatogenic activities. They are also potent antioxidants and protect testicular tissues and sperm cells against oxidative stress (53). Incubation with glucose only significantly (*p* < 0.05) elevated testicular level of sodium (Na), with concomitant decreased levels of magnesium (Mg), sulfur (S), potassium (K), calcium (Ca), and iron (Fe) (Figure 11). These levels were significantly (*p* < 0.05) reversed following incubation with African walnut oil. Ca and Mg have been reported for their roles in spermatogenesis, sperm maturation, and motility (54). The spermatogenic roles of Na and K include semen production, sperm capacitation, motility, fertilization capacity, and membrane potential but toxic at higher concentrations (53). Fe is a non-enzymic antioxidant but a pro-oxidant at higher concentrations (55), while sulfur improves spermatogenesis (56).

### 3.11 Fatty acid profiles

**Figure 12:**
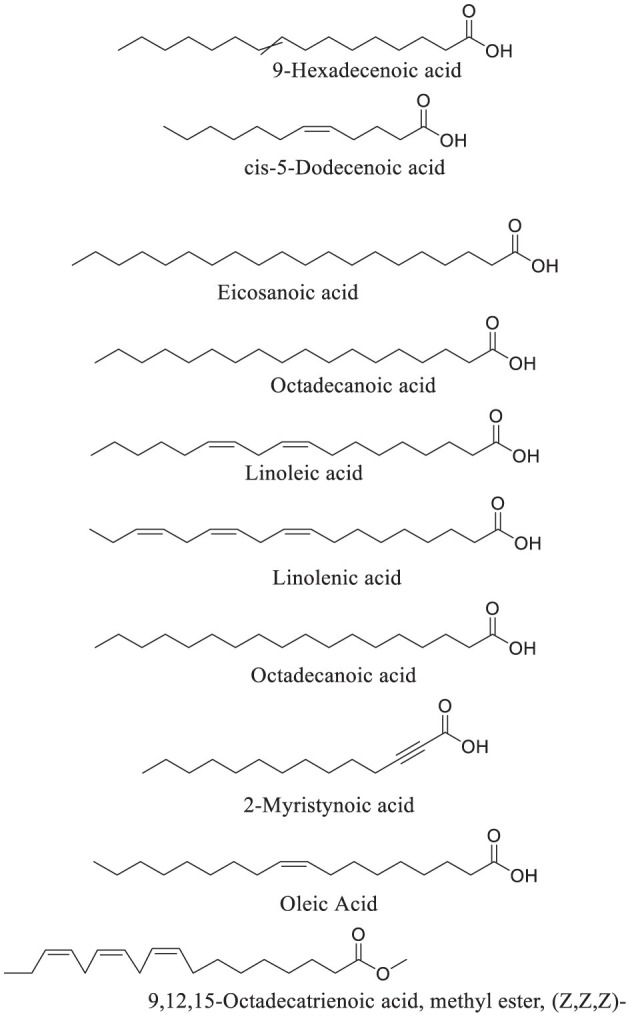
GC-MS identified fatty acid constituents of African walnut oil.

GC-MS analysis of African walnut oil revealed linoleic acid and linolenic acid as the predominant fatty acids (Figure 12; Table 1). Other fatty acids identified were 9-hexadecenoic acid, eicosanoic acid, cis-5-dodecenoic acid, octadecanoic acid, 9,12,15-octadecatrienoic acid, methyl ester, (z,z,z)-, octadecanoic acid, 2-myristynoic acid, and oleic acid (Figure 12; Table 1). Linoleic acid and linolenic acid, commonly known as omega-6 and omega-3 fatty acids, are essential fatty acids which are parts of the cell membrane structure, precursors to bioactive lipid mediators, and energy source. They are also constituents of sperm cells and are linked to potent antioxidant activities, improved sperm counts, motility, and morphology (56–58).

**Table 1:** GC-MS identified fatty acid constituents of African walnut oil.

**Compounds**	**Relative abundance (%)**
9-Hexadecenoic acid	0.1
Eicosanoic acid	4.1
cis-5-Dodecenoic acid	0.14
Octadecanoic acid	0.13
9,12,15-Octadecatrienoic acid, methyl ester, (Z,Z,Z)-	0.26
Linoleic acid	39.03
Linolenic acid	42.89
Octadecanoic acid	11.63
2-Myristynoic acid	0.13
Oleic Acid	0.27

### 3.12 Molecular docking

**Figure 13:**
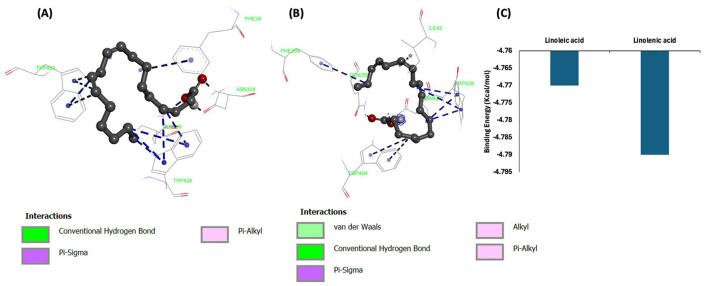
Molecular interactions of **(A)** linoleic acid and **(B)** linolenic acid with GLUT4. **(C)** Binding energy.

Molecular docking of linoleic acid and linolenic acid with GLUT4 showed potent molecular interactions as depicted by their binding energies, with linolenic acid showing a higher interaction (Figures 13A–C). The interactions can be attributed to the formation of hydrogen bond and hydrophobic interactions with amino acids of the target protein (GLUT4) as shown in Table 2. These potent molecular interactions corroborate the ability of the oil to elevate GLUT4 level. They also correlate with previous studies on the ability of these essential fatty acids to stimulate translocation of GLUT4, thereby promoting glucose uptake (59–61).

**Table 2:** Interactive bonds of linoleic acid and linolenic acid with GLUT4.

**Protein**	**Compounds**	**Hydrogen bonds (distance Å)**	**Hydrophobic interaction**
GLUT4	Linoleic acid	• GLN299 (1.66417) • ASN304 (1.95979) • ASN304 (1.9458)	• TRP404 (x4) • TRP428 (x5) • PHE38
	Linolenic acid	• GLN298 (2.12506) • ASN427 (2.1782)	• ILE42 • PHE395 • TRP404 (x2) • TRP428 (x3)

## 4 Conclusion

Taken together, these results suggest the ability of African walnut oil to improve testicular glucose metabolism and steroidogenesis and can be explored in the management of male infertility. This is evident by its ability to stimulate glucose uptake, promote steroidogenesis, glycolysis and the glyoxalase system, and improve energy metabolism while suppressing polyol pathway and glucogenesis. These activities can be attributed to its fatty acid constituents, especially the omega-3 and omega-6 fatty acids. The limitation of the presentation is the lack of *in vivo* studies to back these findings. Thus, preclinical and clinical studies will be required to further decipher the molecular mechanism underlying the oil's effect on the male reproductive system.

## Data Availability

The original contributions presented in the study are included in the article/supplementary material, further inquiries can be directed to the corresponding author.
